# Control of Murine Cytomegalovirus Infection by γδ T Cells

**DOI:** 10.1371/journal.ppat.1004481

**Published:** 2015-02-06

**Authors:** Sabrina Sell, Monika Dietz, Andrea Schneider, Rafaela Holtappels, Michael Mach, Thomas H. Winkler

**Affiliations:** 1 Nikolaus-Fiebiger-Zentrum für Molekulare Medizin, Department Biologie, Friedrich-Alexander-Universität Erlangen-Nürnberg, Erlangen, Germany; 2 Institut für Klinische und Molekulare Virologie, Friedrich-Alexander-Universität Erlangen-Nürnberg, Erlangen, Germany; 3 Institut für Virologie und Forschungszentrum für Immuntherapie (FZI), Universitätsmedizin der Johannes Gutenberg-Universität Mainz, Mainz, Germany; University of Minnesota Medical School, UNITED STATES

## Abstract

Infections with cytomegalovirus (CMV) can cause severe disease in immunosuppressed patients and infected newborns. Innate as well as cellular and humoral adaptive immune effector functions contribute to the control of CMV in immunocompetent individuals. None of the innate or adaptive immune functions are essential for virus control, however. Expansion of γδ T cells has been observed during human CMV (HCMV) infection in the fetus and in transplant patients with HCMV reactivation but the protective function of γδ T cells under these conditions remains unclear. Here we show for murine CMV (MCMV) infections that mice that lack CD8 and CD4 αβ-T cells as well as B lymphocytes can control a MCMV infection that is lethal in RAG-1^-/-^ mice lacking any T- and B-cells. γδ T cells, isolated from infected mice can kill MCMV infected target cells in vitro and, importantly, provide long-term protection in infected RAG-1^-/-^ mice after adoptive transfer. γδ T cells in MCMV infected hosts undergo a prominent and long-lasting phenotypic change most compatible with the view that the majority of the γδ T cell population persists in an effector/memory state even after resolution of the acute phase of the infection. A clonotypically focused Vγ1 and Vγ2 repertoire was observed at later stages of the infection in the organs where MCMV persists. These findings add γδ T cells as yet another protective component to the anti-CMV immune response. Our data provide clear evidence that γδ T cells can provide an effective control mechanism of acute CMV infections, particularly when conventional adaptive immune mechanisms are insufficient or absent, like in transplant patient or in the developing immune system *in utero*. The findings have implications in the stem cell transplant setting, as antigen recognition by γδ T cells is not MHC-restricted and dual reactivity against CMV and tumors has been described.

## Author Summary

Cytomegalovirus is a clinically important pathogen. While infection in hosts with a functional immune system is usually asymptomatic, the virus can cause significant morbidity and mortality in individuals with an immature or suppressed immune system. The virus causes severe clinical complication in transplant recipients and congenital CMV infections are the most common infectious cause of neurological disorders in children. Multiple layers of innate and adoptive immunity are involved in the control of CMV and single deficiencies of one immune cell type can be compensated by other immune cells. Expansions of γδ T lymphocytes, which are regarded as innate-like cells with adaptive-like potential, have been shown to be associated with CMV infections in human transplant patients and neonates. Their role in protective immunity against CMV has been unclear, however. Here we show direct evidence in the murine CMV model (MCMV) that γδ T lymphocytes can provide protection against a lethal MCMV infection in the absence of any other cells of the adoptive immune system. Upon infection, γδ T lymphocytes undergo a significant expansion and a prominent and long-lasting phenotypic change. These findings have implications for the development of new cellular therapy regimens in CMV infections in the transplant setting that should be evaluated in the future.

## Introduction

Cytomegaloviruses (CMV) and their respective hosts have co-evolved over long periods of time. During this co-evolution the virus adapted perfectly to the respective host defense systems and vice versa. As a result, virus replication during primary infection is effectively controlled by a multilayered, in large parts redundant, innate as well as adaptive immune response and development of symptoms or disease is prevented [1–3].

In contrast, in individuals with a compromised or immature immune system human CMV (HCMV) remains as a significant pathogen. Congenital HCMV infection, for example, is the leading infectious cause of brain damage and sensorineural hearing loss in children [4]. Primary and recurrent HCMV infection also causes significant morbidity and mortality in transplant patients e.g. following hematopoietic cell transplantation [5].

During primary infection of immunocompetent hosts the virus evokes a strong innate and adaptive immune response, which ultimately leads to control of viral replication and establishment of latency [6]. In the adaptive cellular immune response CD8^+^ and CD4^+^ T cells are involved. Activation of antiviral CD8^+^ T cells is considered to be particularly important in this respect. Work with MCMV has shown that antiviral CD8^+^ T cells can control a primary infection [7] and prevent disease after adoptive transfer in immunocompromised animals [8]. The concept of adoptive transfer of CMV specific CD8^+^ T cells to prevent CMV-related disease was successfully introduced into clinical management of transplant patients [9].

The role of CD4^+^ T cells in protection from CMV infection is less clear. In the MCMV system, CD4^+^ T cells have been shown to be essential for viral clearance from the salivary gland, an important anatomical site for prolonged CMV shedding and transmission [10]. The antiviral effect is most probably mediated by IFN-γ secretion of activated CD4^+^ T cells [11]. In transplant patients a multifunctional effect of CD4^+^ T cells has been shown. They provide essential support for CD8^+^ T cell memory, secrete various cytokines and even kill infected cells [12].

Lastly, the adaptive humoral immune response is also involved in control of viral replication.

Immunodeficient RAG^-/-^ mice are protected against MCMV infection by passive transfer of polyclonal or monoclonal antibodies from infected donors or MCMV specific memory B cells [13–16].

Despite the large body of literature on protective capacity of individual immune effector functions that contribute to the control of CMV, none of the innate or adaptive immune functions seems to be essential for virus control. For example, in the MCMV model, long-term depletion of CD8^+^ T cells showed that CD8^+^ T cells are not required for clearance of a primary CMV infection and viral latency was initiated with similar kinetics in animals lacking CD8^+^ T cells [17]. Similar results were obtained following depletion of the CD4^+^ T-cell subset [10]. Likewise, the course of the primary CMV infection is similar in fully immunocompetent mice and mice lacking antibody [15]. On the other hand, RAG^-/-^ or SCID mice succumb rapidly to the infection, demonstrating that the complete lack of an adaptive immune response is incompatible with virus control [14, 18].

It is unclear whether the redundancy in immunological control of CMV is universal, i.e. the loss of any antiviral immune effector function can be compensated by others or whether the redundancy is restricted to a certain set of immune cells or antiviral effector function. The definition of critical components of the immune response that cannot be compensated for by others will be important to understand the complex immune control of this virus and may be valuable for the clinical management of the infection in situations where the immune system is not fully functional.

Here we systematically explored the contribution of individual cell types of the adaptive immune system to virus control. Surprisingly, our data showed that mice lacking CD4^+^ and CD8^+^ T cells as well as B cells still can control the virus for long periods of time. Protection in these animals was found to rely on CD3^+^CD8^−^CD4^−^ γδ T cells. Depletion of CD3^+^ T cells in mice lacking B cells abolished protection from CMV infection and most importantly adoptive transfer of γδ T cells into RAG^-/-^ mice provided long term protection from the otherwise lethal course of the infection. The identification of γδ T cells as a protective component in the anti-CMV response adds another layer to the complex immune control of this virus which may have clinical implications in the transplant setting since the function of γδ T cells is not restricted by the MHC complex.

## Results

### Mice without CD8^+^ and CD4^+^ T cells as well as B cells but not without CD3^+^ T cells control CMV infection

**Figure 1:**
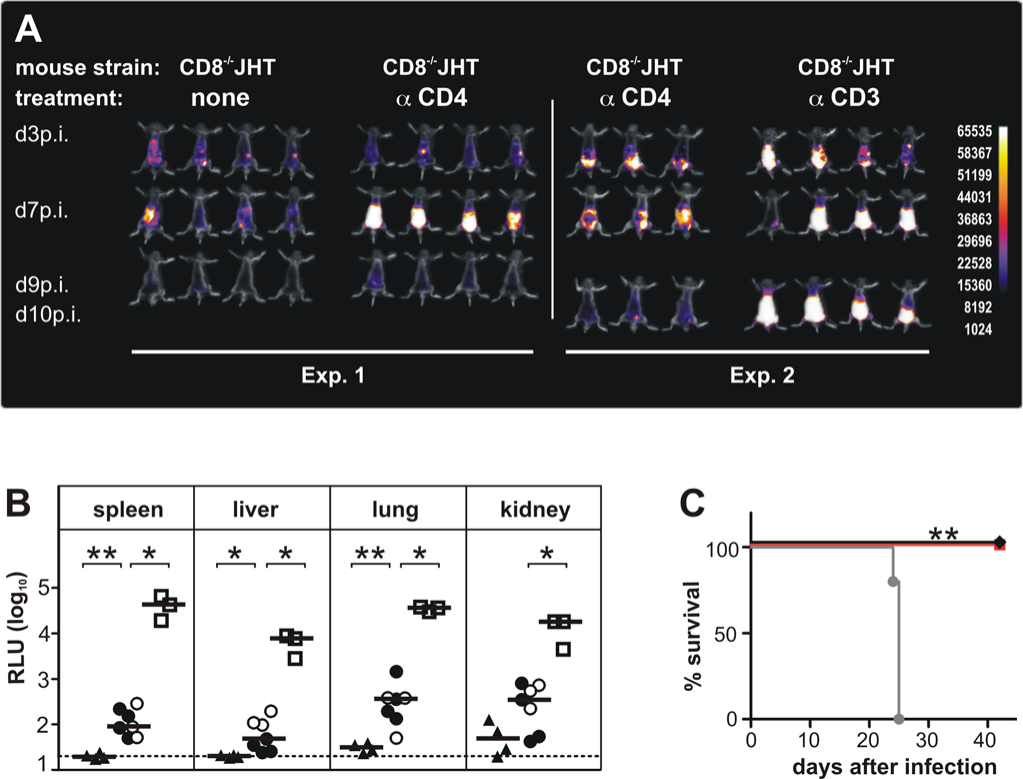
CD3^+^ T cells control MCMV infection in mice depleted of CD4^+^ and CD8^+^ T-lymphocytes and B cells. (A) In two separate experiments CD8^-/-^JHT mice either untreated or treated with antibodies against CD4 or CD3 were infected with 10^5^ pfu MCMV. *In vivo* imaging was performed on the days indicated. Images were obtained from a 120 sec acquisition. The pseudocolor scale shows relative photon flux for each image. Representative data from 3 individual experiments. (B) Viral load per 30 μg organ was determined at day 13 p.i. by a luciferase-based assay. Each value in the graph corresponds to an individual animal, one animal died before day 13 p.i. triangles: CD8^-/-^JHT; circles: anti-CD4 treated CD8^-/-^JHT; squares: anti-CD3 treated CD8^-/-^JHT. Data in open or closed symbols were obtained in two separate experiments from mice displayed in Fig. 1A. Horizontal bars represent the median, dotted lines mark the background. RLU: relative light units. P < 0.05; Mann-Whitney test. Representative data from 3 individual experiments. (C) Survival of CD8^-/-^JHT (black), anti-CD4 treated CD8^-/-^JHT (red) and RAG^-/-^ mice (grey) (n = 5; P < 0.005; Mantel-Cox Test). Representative data from 2 independent experiments.

In a first set of experiments we analyzed the potential effect of a combined absence of CD8^+^ T cells as well as antibodies for the course of a primary MCMV infection. To this end CD8^-/-^JHT mice (S1 Fig. and S1 Table) were infected with 1×10^5^ pfu of a luciferase–expressing MCMV suitable for *in vivo* detection of virus infected cells [14]. In CD8^-/-^JHT mice, luciferase activity was detected at day 3 post infection (p.i.) and day 7 p.i. which fell to background levels at day 9 p.i. (Fig. 1A). In these mice, the course of acute infection was slightly prolonged as compared to animals in which either cell type was lacking individually or as compared to immunocompetent C57BL/6 animals [14]. Thus, in mice with a combined lack of CD8^+^ T cells and B cells acute MCMV infection can be controlled.

To analyze a potential contribution of CD4^+^ T cells in animals lacking CD8^+^ T cells and B cells for the course of the infection, CD8^-/-^JHT animals were treated with 250 µg of mab YTS 191 on day -1, 3 and 8 p.i. [15]. Absence of CD4^+^ T cells was confirmed by flow cytometry (S2 Fig.). Following infection, the mice showed a markedly higher bioluminescence signal compared to CD8^-/-^JHT animals at day 7 p.i. (Fig. 1A). However, at day 9 p.i. the signal was greatly reduced indicating control of virus replication in the animals.

The control of infection in animals lacking both CD8^+^ and CD4^+^ T cells as well as B cells was surprising given the fact that RAG^-/-^ animals, which do not contain functional T and B cells, are not capable of controlling MCMV [14, 18]. Potential T cell subsets responsible for protection would include CD3^+^ NKT cells, CD4/CD8-double negative (DN) αβ T cells or γδ T cells.

**Figure 2:**
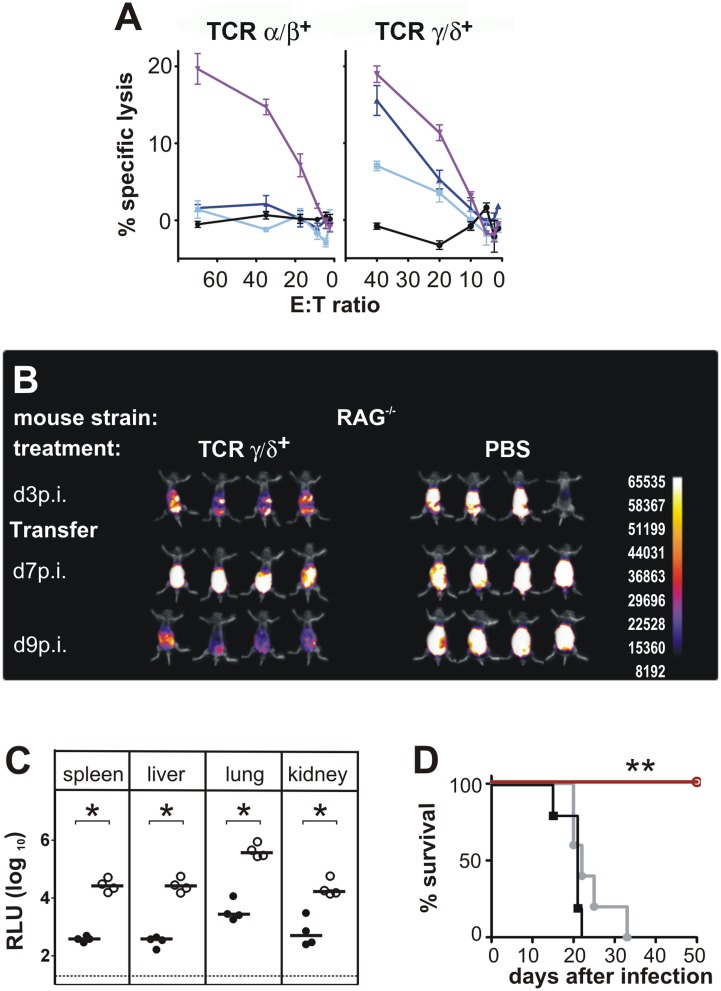
Functional activity of CD3^+^DN T cells. (A) CD3^+^DN TCRαβ^+^ or TCRγδ^+^ effector cells were isolated from CD8^-/-^ JHT mice 4 weeks after ip infection and were titrated in log_2_ steps. Mouse embryonic fibroblasts (MEF) were infected with MCMV Smith and used as target cells in a standard ^51^Cr-release assay. Lysis of target cells was assayed at the effector-cell-to-target-cell (E/T) ratios indicated. The mean percentage of specific lysis of target cells is shown, calculated from triplicate assay cultures. Black: uninfected MEF, light blue: late phase-infected MEF, dark blue: early phase-infected MEF, purple: TCR-independent stimulation of T cells by the CD3ε-producing hybridoma cell line 145–2C11. Representative data from 2 individual experiments. (B) RAG^-/-^ mice were transferred 3 days after infection with 800,000 γδ T cells from infected CD8^-/-^ JHT donor mice. *In vivo* images were obtained as in Fig. 1. Representative data from 4 experiments are shown. (C) Viral load per 30 µg organ was determined at day 12 p.i. Each value in the graphs corresponds to an individual animal (closed circles: γδ T-cell transferred animals; open circles: PBS controls); horizontal bars represent the median. Dotted lines mark the background. (P < 0.05, Mann-Whitney Test). Representative data from 4 individual experiments. (D) Survival of RAG^-/-^ mice adoptively transferred with 200,000 γδ T cells from naive (black) or infected (red) donors or PBS treated (grey) (P < 0.005; Mantel-Cox Test). Representative data from 4 experiments.

To test whether DN T cells were involved in control of MCMV infection, CD3^+^ cells were depleted in CD8^-/-^JHT animals. Depletion was confirmed by flow cytometry (S2 Fig.). Following infection, anti-CD3 antibody treated animals showed markedly enhanced bioluminescence compared to untreated or anti-CD4 antibody treated CD8^-/-^JHT mice at days 7 and 9 p.i., indicating loss of control of virus replication (Fig. 1A). The bioluminescence signals from anti-CD3 treated animals were comparable to infected RAG^-/-^ mice which exhibit a continuously increasing bioluminescence signal during the first 10 days p.i. ([14] and Fig. 2B).

To correlate the *in vivo* bioluminescence data with virus titers in individual organs, animals were sacrificed at day 13 p.i. and the virus load was determined in selected organs, using a luciferase-based assay. Viral organ titers supported the data from the *in vivo* imaging. Very low levels of viral titers were observed in CD8^-/-^JHT mice (Fig. 1B). In CD4^+^ T cell-depleted animals, the viral titers in organs were slightly elevated at day 13 p.i. compared to CD8^-/-^JHT animals, indicating that the control of the infection during simultaneous absence of CD8^+^, CD4^+^ and B cells is not as effective as in CD8^-/-^JHT mice (Fig. 1B). Viral titers in CD3-depleted CD8^-/-^JHT mice were significantly increased and comparable to titers obtained in RAG^-/-^ animals (Fig. 1B and Fig. 2C).

In survival experiments, CD8^-/-^JHT mice either in the presence or absence of CD4^+^ T cells exhibited long-term control of the infection further indicating efficient virus control in the absence of CD8^+^/CD4^+^ T cells as well as B cells (Fig. 1C). In contrast, and in accordance with our previous results [14], RAG^-/-^ animals succumbed to the infection between days 21–25 (Fig. 1C).

Taken together, the results indicated that mice lacking B cells, CD8^+^ and CD4^+^ T cells are still able to control a primary MCMV infection. The protecting cell type in these animals most probably exhibits a CD3^+^ DN phenotype since protection was lost following depletion of CD3^+^ T cells.

### Functional activity of CD3^+^CD4^−^/CD8^−^ T cells

To test whether DN T cells have direct antiviral activity, a standard *in vitro* cytotoxicity assay was performed with αβ and γδ positive DN T cells in a functional chromium release assay *ex vivo* without restimulation of cells. To this end, DN TCRαβ^+^ and γδ T cells were purified from spleen and lymph nodes of infected CD8^-/-^JHT mice 4 weeks after infection and tested for their cytolytic activity. The results shown in Fig. 2A revealed that both cell types were capable of mediating cytotoxicity in a CD3ε-redirected cytotoxicity assay [19]. DN TCRαβ^+^ T cells showed negligible lysis of both early and late infected mouse fibroblast target cells (MEF). In contrast, the γδ T cell fraction specifically lysed both types of MCMV infected MEF (Fig. 2A). Early infected target cells were lysed to a higher degree than late infected target cells, indicating that infected cells express antigens that are target of γδ T cell cytotoxicity during all stages of infection but especially in the early phase after infection.

To analyze whether the ability of γδ T cells to kill infected targets *in vitro* correlates to a protective potential of these cells *in vivo*, an adoptive cell transfer approach was used. To this end γδ T cells were purified from MCMV infected CD8^-/-^JHT donor animals. Purity assessed by flow cytometry reached >99%. In the experiment shown in Fig. 2B 800,000 cells/animal were infused into RAG^-/-^ recipients that had been infected three days before. Animals that received γδ T cells 3 days after infection, exhibited increased bioluminescence signals between day 3 and day 7 p.i. that was comparable to the control group of mice that received no T cells. However, while in the control group the signal intensity further increased between day 3 and day 7 p.i., the bioluminescence was markedly reduced in γδ T cell treated mice at day 9 p.i., indicating control of virus replication. When the viral load in organs was assessed at day 12 p.i., γδ T cell-substituted mice showed a significantly lower viral titer compared to untreated RAG^-/-^ animals (Fig. 2C). In a number of independent experiments using γδ T cell numbers for adoptive transfer ranging from 200,000 to 800,000 per recipient significant reductions of viral load in all organs analyzed were observed. The absence of CD4^+^ T cells in the recipient animals 12 days after infection was confirmed by flow cytometry (S2 Fig.), strongly suggesting that the antiviral effect of the adoptively transferred cell population rests on γδ T cells. γδ T cells provided long-term protection, as RAG^-/-^ mice that received 200,000 γδ T cells from MCMV-immune donors survived the infection for at least 50 days (Fig. 2D). In contrast, control RAG^-/-^ animals showed a significantly shorter survival after infection (mean survival time 22 days). Interestingly, γδ T cells that were isolated from naive animals were not able to protect the animals from the lethal course of the infection (Fig. 2D).

To extend this finding and to investigate whether γδ T cells from wildtype C57Bl/6 mice can protect from MCMV infection after adoptive transfer we transferred 400,000 γδ T cells obtained from the spleen of C57Bl/6 mice that were infected with MCMV at least 4 weeks before or from uninfected C57Bl/6 mice. The protective capacity of γδ T cells from infected and uninfected wildtype mice was much more variable, presumably due to the extended time needed for the cell purification from a large number of donor animals. The results show that γδ T cells from infected wildtype mice could clearly reduce the virus load after adaptive transfer into RAG^-/-^ mice and that they provided better protection than γδ T cells from naïve donor mice. The results reached significance only for the lung, however (S3 Fig.).

In addition we performed infections with TCRα^-/-^ mice that completely lack αβ T cells. In these experiments we used MCMV157luc in which the MCK-2 mutation was repaired [20] and 10^6^ pfu virus was injected i.v. This virus and infection dose and route caused an even more severe infection in RAG-1^-/-^ mice and infected mice had to be euthanized 12 days after infection (S4 Fig.). TCRα^-/-^ mice, however, were able to control virus infection within 2 weeks with only residual virus infection detectable in the salivary glands (S4 Fig.). The protection of TCRα^-/-^ mice was long-lasting as infected mice survived for more than 5 month without any signs of sickness.

In addition we analyzed whether a response after a secondary high dose infection with MCMV is improved in TCRα^-/-^ mice. When mice received a second infection at day 21 after the primary infection with 10^6^ pfu MCMV157luc i.v., virus spread was controlled immediately and efficiently as hardly any increase of bioluminescence was detectable at 10 days after the second infection (S5 Fig.).

Taken together, the results show that γδ T cells are capable of controlling a primary MCMV infection in the absence of additional cells from the adaptive immune system.

### Relevance of γδ T cells for MCMV infection in immunocompetent mice

**Figure 3:**
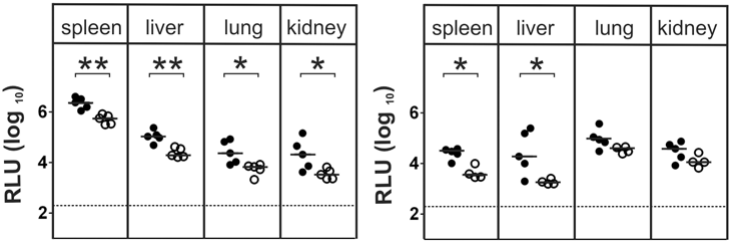
γδ T cells contribute to virus control in immunocompetent animals. Viral load in the organs of TCRδ^-/-^ (closed circles) and C57BL/6 wt (open circles) mice was determined at day 3 p.i. (left) and day 5 p.i. (right). Each value in the graphs corresponds to an individual animal; horizontal bars represent the median (P < 0.05, Mann Whitney Test). Representative data from 2 individual experiments.

The results so far indicated a protective capacity of γδ T cells in the absence of additional components of the cellular and humoral immune response. The question arises, whether γδ T cells have also a role in the antiviral response in fully immunocompetent hosts. We addressed this question by using TCRδ^-/-^ mice. Three days after MCMV-infection, TCRδ^-/-^ mice showed significantly higher viral titers in all tested organs as compared to C57BL/6 mice (Fig. 3). Five days after infection, the difference was less pronounced, being significant only in spleen and liver. These data indicated that during the early phase of infection, the lack of αβ T cells in otherwise fully immunocompetent animals results in higher viral titers.

### Cytokine production of γδ T cells and their functional relevance after MCMV- infection

**Figure 4:**
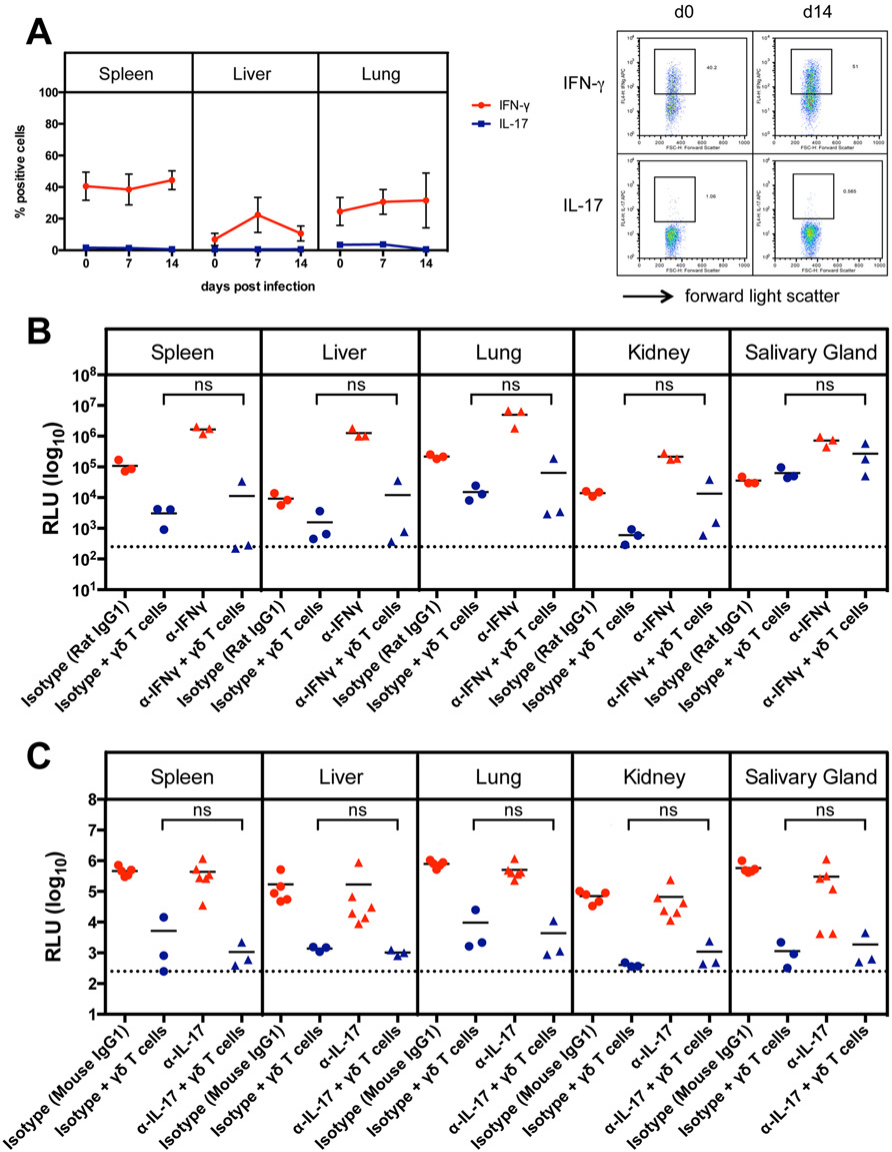
Cytokine production of γδ T cells after MCMV infection. (A) Intracellular cytokine production of γδ T cells during MCMV infection in CD8^-/-^JHT mice. The mean and SD values of the percentages of positive γδ T cells for IFNγ and IL-17 are displayed (n = 5). A representative FACS plot is shown on the right. (B) Influence of neutralizing anti-IFNγ monoclonal antibodies on the protective capacity of adoptively transferred γδ T cells in RAG^-/-^ mice. Viral load per 30 µg organ was determined at day 18 p.i. Lines represent mean values. Horizontal bars represent the mean values (ns: not significant, Mann Whitney Test). Representative data from 2 experiments. (C) Influence of neutralizing anti-IL-17 monoclonal antibodies on the protective capacity of adoptively transferred γδT cells in RAG^-/-^ mice. Viral load per 30 µg organ was determined at day 18 p.i. Lines represent mean values (ns: not significant, Mann Whitney Test).

As the production of interferon-γ (IFNγ) and interleukin 17 (IL-17) by γδ T cells can influence the outcome of an immune response [21] we analyzed the intracellular IFNγ and IL-17 production during infection in spleen, liver and lung of CD8^-/-^JHT mice by flow cytometry. As shown in Fig. 4A a high frequency of IFNγ-producing γδ T cells was observed that remained relatively constant in spleen and liver during the infection. In the liver on day 7 after infection a transient increase of IFNγ-producing γδ T cells was observed. IL-17 producing γδ T cells were very rare in these organs, as described before [22], and the frequency did not change during MCMV infection (Fig. 4A).

To examine whether IFNγ or IL-17 play a role in viral control, we performed adoptive transfer experiments as described above using RAG^-/-^ recipients and sorted γδ T cells from previously infected CD8^-/-^JHT mice. Neutralizing antibodies against IFNγ or IL-17 or isotype control antibodies were given to the animals before and during the time course of the infection. Neutralizing anti-IFNγ antibodies significantly elevated the viral load in RAG^-/-^ control animals that did not receive γδ T cells presumably because NK cell-derived IFNγ, which provided some protection effect, was blocked (Fig. 4B, red symbols). Importantly, however, the protective capacity of γδ T cells was not significantly influenced by neutralizing anti-IFNγ antibodies (Fig. 4B, blue symbols). Neutralizing IL-17 antibodies had no effect on the viral load in RAG^-/-^ mice and did not influence the protective capacity of adoptively transferred γδ T cells (Fig. 4C).

### Numerical and phenotypic changes of γδ T cells after MCMV- infection

The fact that adoptively transferred γδ T cells were able to reduce the viral load in different organs indicated that the protective fraction of the γδ T cells is widely distributed in the organism, most probably via the bloodstream. In the blood and secondary lymphoid system γδ T cells represent only a minor proportion of lymphocytes but they are much more frequent in epithelial-rich organs [23].

**Figure 5:**
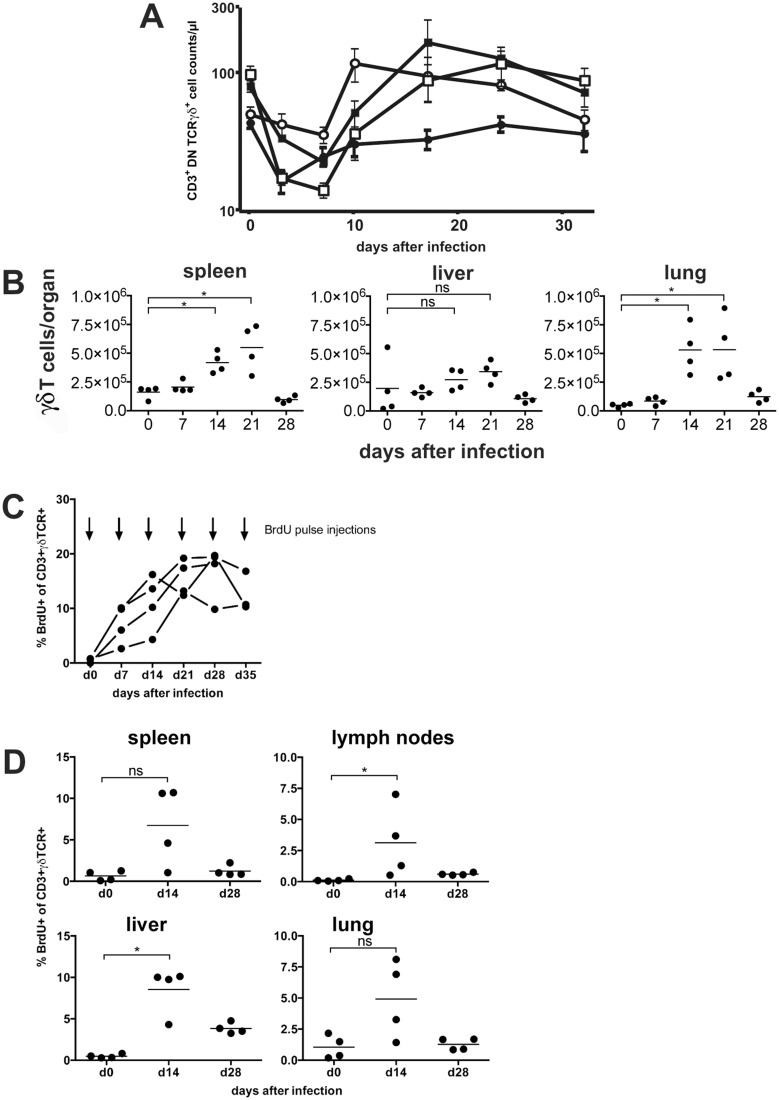
γδ T cells proliferate in the peripheral blood and in target organs after infection. (A) Mice (n = 4) from different mouse strains were infected with 10^5^ pfu MCMV. Blood samples were taken from all animals on the days indicated, stained with fluorescence conjugated anti-CD3, anti-CD4, anti-TCR and anti-TCRγδ antibodies and quantified by flow cytometry using counting beads. Values represent mean values ±SEM. (filled circles: C57BL/6; open circles: CD8^-/-^; filled squares: CD8^-/-^JHT; open squares: anti-CD4 treated CD8^-/-^JHT). Representative data from 2 individual experiments are shown. (B) Spleen, liver and lung tissue was obtained from infected mice (n = 4). TCRγδ positive cells were quantified by flow cytometry using counting beads. Absolute numbers of CD3^+^ TCRγδ^+^ cells per organ from individual animals (dots) and medians (bars) are shown. Data from one experiment are shown (* p<0.05, Mann Whitney Test). (C) CD8^-/-^JHT mice (n = 4) were pulse labeled by weekly BrdU injections and BrdU uptake was analyzed 6 hours after pulse BrdU injections in the peripheral blood. The percentage of BrdU-positive peripheral blood γδ T cells was determined by flow cytometry. (D) CD8^-/-^JHT mice (n = 4) were pulse labeled by BrdU injections before infection or 2 weeks and 4 after infection. BrdU uptake was analyzed 6 hours after BrdU injections. The percentage of BrdU-positive γδ T cells was determined by flow cytometry for the different organs labeled in the graphs (* p<0.05, Mann Whitney Test).

γδ T-cell populations were analyzed in peripheral blood, spleen, liver and lung of CD8^-/-^JHT mice after MCMV infection. Blood samples were taken from different mouse strains on days 0, 3, 7, 10, 17, 24 and 32 p.i. and stained for CD3/4/8, TCRαβ and TCRγδ. Absolute cell counts of γδ T cells were quantified by flow cytometry (Fig. 5A). Between the tested mouse strains, namely C57BL/6, CD8^-/-^, CD8^-/-^JHT and anti-CD4 treated CD8^-/-^JHT no substantial differences could be detected. γδ T-cell counts were reduced substantially on days 3 and 7 after infection in all strains. Following this general lymphopenia, most likely caused by the early production of type I interferon upon virus infection [24], the population started to recover to preinfection levels 10 days p.i. and remained relatively constant until the end of the experiment at day 32. Within spleen, liver and lung the numbers of γδ T cells moderately increased at day 14 and 21 after infection and returned to preinfection levels on day 28 (Fig. 5B).

To analyze whether γδ T cells proliferate *in vivo* after MCMV infection, short-term BrdU pulse experiments were performed at different time points after infection of CD8^-/-^JHT mice. Six hours after BrdU injection less than 1% of γδ T cells in the peripheral blood incorporated BrdU in uninfected mice, showing that the vast majority of γδ T cells are non-cycling *in vivo* (Fig. 5C). In mice that were infected with MCMV a significant fraction of γδ T cells present in the peripheral blood incorporated BrdU 6 hours after injection showing that up to 20% of γδ T cells proliferated *in vivo* in response to MCMV infection. In the organs of infected mice γδ T cells also showed significant proliferation 2 weeks after infection in lymphnodes and liver. Whereas proliferation normalized 4 weeks after infection in spleen, lung and peripheral lymphnodes, in the liver elevated percentages of proliferating γδ T cells are noticeable (Fig. 5D). These data clearly showed that a significant fraction of γδ T cells proliferate in response to MCMV infection *in vivo*.

**Figure 6:**
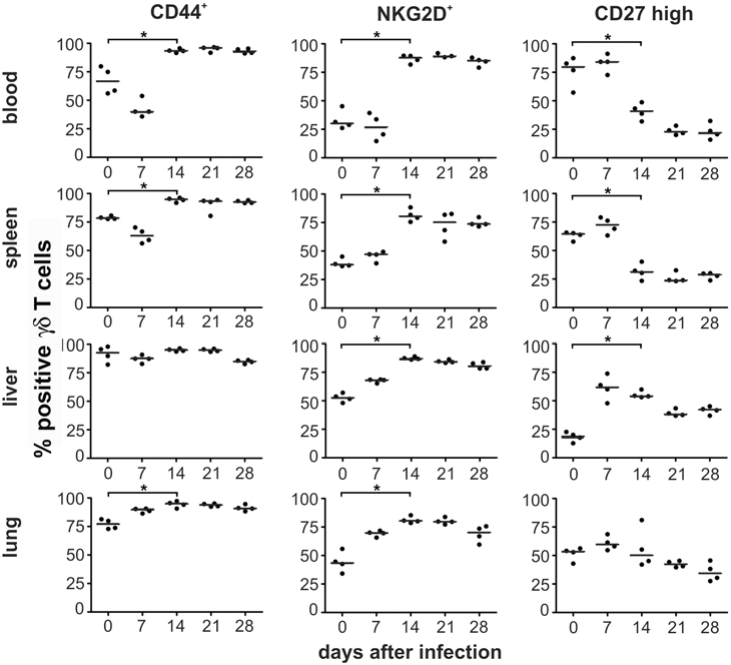
Phenotypical changes of γδ T cells after infection. Blood samples and mononuclear cells from organs of infected CD8^-/-^JHT mice were stained with anti-CD3, anti TCRγδ, anti-CD44, anti-NKG2D and anti-CD27 antibodies. The percentage of total CD3^+^ TCRγδ^+^ cells of individual animals (dots) and medians (bars) are shown. Representative data from 3 individual experiments (* p<0.05, Mann Whitney Test).

To phenotypically characterize the γδ T-cell population after infection we determined several known markers for γδ T cells in blood, spleen, liver and lung. As depicted in Fig. 6, the frequency of CD44^+^ γδ T cells increased in all organs 14 days after infection, suggesting considerable and long lasting activation of γδ T cells in infected animals. The increased frequency of CD44^+^ γδ T cells coincided with a switch in the expression pattern of NKG2D, a lectin-like stimulatory receptor originally identified in NK cells [25] and CD27, a costimulatory receptor of the TNF-receptor superfamily [26], on the majority of γδ T cells in all organs analyzed (Fig. 6). Whereas before infection and during the early phase after infection (days 0–7 p.i.) the majority of γδ T cells were NKG2D- and CD27^high^ a switch to NKG2D^+^ CD27^low^ was observed at later times after infection in the majority of cells (Fig. 6 and S6 Fig.). This switch of the phenotype remained constant during the observation period until day 28 p.i., when MCMV infection was undetectable by bioluminescence. Thus, MCMV infection induced a significant and long-lasting activation and change in the phenotype of the majority of γδ T cells in secondary lymphoid organs and tissues in which MCMV infection is prominent.

### Clonotypic expansion of Vγ1 and Vγ2 cells in target organs of persistent MCMV infection

To determine whether in MCMV infected mice subpopulations of clonally restricted γδ T cells expand, as it has been described for human γδ T cell subpopulations [27] we first determined the Vγ usage by flow cytometry using currently available antibodies. As expected Vγ1 and Vγ4 cells (according to the nomenclature of Heilig et al. [28]) contributed to over 80% of γδ T cells in spleen, lymph nodes, lung and liver whereas Vγ5 and Vγ7 cells were hardly detectable in these organs. The only major change in the relative contribution of TCRγ subpopulations was an increase of Vγ1 cells from approximately 50% to up to 80% of all γδ T cells particularly in liver and lung of infected mice at 21 and 28 days after infection in CD8^-/-^JHT and TCRα^-/-^ mice.

**Figure 7:**
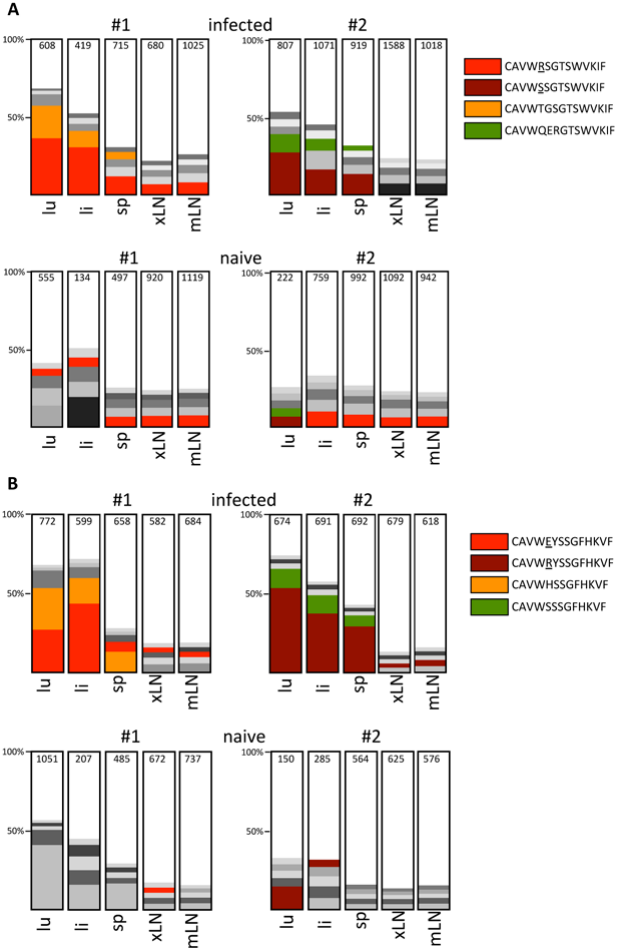
Analysis of the Vγ1 and Vγ2 repertoire by high throughput 454 sequencing. Analysis of expanded Vγ1 (A) and Vγ2 (B) clonotypes in infected (d28 post infection) and uninfected CD8^-/-^JHT mice. The representations of the five most abundant clonotypes as defined by identical CDR3 regions are depicted for lung (lu), liver (li), spleen (sp), peripheral lymph nodes (xLN) and mesenteric lymph nodes (mLN) in 2 individual mice for each group in the bar graph. Clonotypes found in different organs and mice are represented with the same color and the CDR3 sequences are displayed in the legend. The total numbers of sequence reads obtained for each organ and Vγ gene is presented in the bars.

To evaluate in detail whether the long-lasting activation and phenotypic change of γδ T cells correlates with a more focused Vγ usage we determined the Vγ repertoire in 5 different organs of two infected (d28) and two uninfected CD8-JHT mice by 454 sequencing of Vγ-Cγ amplicons obtained from cDNA from sorted γδ T cells. As expected, sufficient numbers of reads from lung, liver and lymphoid tissues were obtained only for Vγ1, Vγ2, Vγ4 and Vγ6 recombinations. When all amplicons were analyzed for recurrent recombinations we found one major CDR3 clonotype for Vγ4 and Vγ6 present in all organs, constituting up to 60% and 90% of the respective Vγ4 and Vγ6 genes. Importantly, no major difference was observed between infected and uninfected mice (S7 Fig.). Within Vγ1- and Vγ2-products we observed much more diversity particularly in lymphoid organs of uninfected mice. The five most prominent CDR3 clonotypes constitute less than 25% of the whole repertoire (Fig. 7). For the infected mice, however, a clear expansion of Vγ1- and Vγ2- clonotypes was found particularly in lung and liver (Fig. 7). In both mice 2 dominant clonotypes were found expanded in lung and liver and to a lesser extend these clonotypes can also be detected in the spleen of the same animal. In peripheral and mesenteric lymph nodes these clonotypes were not particularly expanded, however. Together, these results suggest a long lasting and focused accumulation of clonotypically related Vγ1- and Vγ2 cells after MCMV infection in the organs of virus persistence and latency [29].

## Discussion

The control of CMV infections relies on multiple and redundant immune effector functions from the innate and the adaptive immune system [3]. In this report, we provide the first direct evidence that γδ T cells, which are regarded as innate-like cells with adaptive-like potential [30], can provide protection against MCMV infection in the absence of other effector cells of the adaptive immune system, i.e. conventional αβ T cells or B cells. The protection against lethal infection conferred by γδ T cells after adoptive transfer into RAG^-/-^ mice clearly showed some adoptive-like elements as only γδ T cells from MCMV-infected donors could provide protection whereas equal numbers of γδ T cells from uninfected animals were unable to provide significant protection in the RAG^-/-^ mice. The fast and efficient control of MCMV after a high dose secondary infection in TCRα^-/-^ mice can be regarded as an additional evidence for an adaptive and memory-like response of γδ T cells, which shares features to the NK cell response after secondary MCMV infection [31]. Whereas in the case of NK cells the invariant “innate” Ly49H receptor is accountable for the response, our data regarding a focusing of the Vγ1 and Vγ2 repertoire particularly in organs where MCMV is persisting suggest the establishment of a response that is selected for by antigen through the γδ antigen receptor formed by VDJ-recombination. It will be interesting to isolate and identify these receptors from infected mice and to generate transgenic mice to study those γδ T cells in a defined way during infection, similar as it has been done for virus specific αβ T cells and B cells.

NKG2D is a C-type lectin found on NK cells and a fraction of γδ T cells and CD8^+^ αβ T cells. The markedly elevated frequency of NKG2D-positive γδ T cells that remained for several weeks after infection in the MCMV-infected animals could suggest an expansion of a subpopulation of NKG2D-positive γδ T cells that is present in relative low frequency in uninfected animals. An alternative explanation for the increased frequency of NKG2D-positive γδ T cells after infection is that it is merely a reflection of the activated state of the entire γδ T-cell population. It has been shown that NKG2D-expression is upregulated on all CD8^+^ T cells upon activation after anti-TCR stimulation and on the majority of antigen-specific CD8^+^ cells after virus infection [25]. The concomitant downregulation of CD27 on NKG2D-positive γδ T cells as well as the upregulation of CD44 further supports the view that the majority of the γδ T-cell population persists in an effector/memory state even after resolution of the acute phase of the infection. CD27-low/negative Vγ9δ2 cells have been shown to belong to the memory and effector/memory compartment in humans [32, 33].

During MCMV infection γδ T cells contribute to protection as early as three days after infection as described here for wildtype mice. This goes hand in hand with the description as pre-activated and pre-programmed cells that offer a first line of defense [21]. We also saw activation of γδ T cells at much later timepoints during the infection corresponding to the second and later response of γδ T cells observed during murine influenza A infection [34]. Interestingly, also in the case of influenza infection, Vγ1^+^ T cells, which are normally localized to lymphoid tissues, dominate the later response. In addition, MCMV as a persisting virus might provide constant stimulation of γδ T cells particularly in the major target organs, causing an increase in cell numbers and phenotypic alterations. In this context it is remarkable that a certain γδ T-cell subpopulation expands in healthy aged HCMV carriers [35].

In general, the antigen specificity of the γδ T-cell receptor (TCR) recognition remains still enigmatic except for a few cases where clear biochemical binding data have been obtained [21]. In the context of recognition of herpes virus infected cells, early studies with γδ T-cell clones suggested specific recognition of a herpes virus glycoprotein I on infected target cells. Very recent data showed that a γδ T-cell clone with dual reactivity towards HCMV infected cells and epithelial tumors binds to a stress-regulated self-antigen, the endothelial protein C receptor [36].

Whereas only two reports so far established a potential function of γδ T cells in rodent CMV infection [37, 38], numerous publications associated γδ T cells with HCMV infections, particularly in renal allograft patients [27, 39, 40], allogenic stem cell transplantation [41] and during HCMV infections of the fetus *in utero* [42]. However, a causal relationship between γδ T-cell responses and protection from CMV disease has not been established. The data presented in this report strongly argue that γδ T cells can provide an effective control mechanism of acute CMV infections, particularly when conventional adaptive immune mechanisms are insufficient or absent. These could include the developing fetus and the period following organ- or allogeneic stem cell transplantation. Recent developments in graft engineering of allogeneic stem cells for transplantation suggest that depletion of TCRαβ positive cells may have advantages over anti-CD3 depletion [43]. In addition to an anti-tumor effect that might be exerted by γδ T cells [36, 44], our findings strongly suggest that the anti-HCMV activities of γδ T lymphocytes could be of benefit for stem cell transplant recipients as suggested previously [41]. Because γδ T lymphocytes are not MHC restricted, adoptive transfer of these lymphocytes from the donor might well represent a new cellular immune-intervention strategy for allogeneic stem cell transplant patients at risk for HCMV infection and reactivation.

## Materials and Methods

### Mice

C57BL/6 mice were obtained from Charles River. C57BL/6 RAG-1^-/-^ (RAG^-/-^) mice were obtained from Irmgard Förster (University Munich) and JHT mice [45] were a gift from Hans-Martin Jäck (Division of Molecular Immunology, University Erlangen-Nürnberg). To obtain CD8^-/-^JHT double knockout mice, CD8α^-/-^ (CD8^-/-^) mice [46] were obtained from The Jackson Laboratory and crossed with JHT mice and double-homozygous offspring were selected.

TCRδ^-/-^ and TCRα^-/-^ mice backcrossed to C57BL/6 were obtained from The Jackson Laboratory. All mice were bred and maintained in the animal facility at the Franz-Penzoldt-Zentrum, University Erlangen under specific pathogen-free environment.

### Viruses

MCMV157luc was described before [14]. Additional viruses were used either containing a repaired mutation in MCK-2 [20] or a deletion of *m126-m129*. The three viruses showed no differences in replication *in vitro* or in experimental outcome. Virus was propagated and purified as described [47]. Virus titer was determined by end-point titration using indirect immunofluorescence on mouse embryonic fibroblasts (MEF) as described [14]. Individual mice were infected intraperitoneally (ip) with 1 × 10^5^ plaque forming units (pfu). In the experiment with TCRα^-/-^ mice infection was done intravenously (i.v.) with 1 × 10^6^ pfu of MCMV157luc in which the MCK-2 mutation was repaired as reported by Jordan et al. [20]. *In vivo* bioluminescence imaging and measurement of organ luciferase activity was done exactly as described [14].

### Antibodies and flow cytometry

After perfusion spleens, lymph nodes, livers and lungs were harvested. Livers and lungs were digested with 2 mg/ml collagenase D (Roche Diagnostics, Mannheim, Germany) and 100 µg/ml DNaseI (Roche Diagnostics) for 60 min at 37°C prior to single-cell suspension. Blood was collected in tubes containing Na heparin (Ratiopharm). After erythrocyte lysis (5 min in 0.15 MNH_4_Cl, 0.02 M HEPES, 0.1 mM EDTA for organs or 10 min in BD FACS Lysing Solution (BD Biosciences) for blood) and FcγR blocking (5 μg/ml rat anti-mouse CD16/CD32; clone 93, eBioscience), cells were incubated in PBS, 2% FCS, 2 mM EDTA for 30 min at 4°C with varying combinations of the following antibodies: CD3-PE (17A2), CD4-PE (GK1.5), TCRγδ-FITC (GL3, BD Biosciences), TCRβ-PE-Cy7 (H57–597, Biolegend, San Diego, CA), CD3-APC (145–2C11), CD4-Alexa Fluor 700 (GK1.5), CD8-Alexa Fluor 700 (53–6.7), CD27-FITC (LG.7F9), NKG2D-PE (CX5), TCRγδ-PerCPeFluor 710 (GL3, eBioscience) and Vγ3/5-FITC (536, BD Biosciences). Antibodies Vγ1-FITC (2.11), Vγ4-FITC (49.2) and Vγ7-FITC (F2.67) were a kind gift from P. Pereira. To determine absolute cell numbers Trucount Beads (BD Biosciences) were added.

FACS analysis was performed on LSRII or FACSCalibur machines (Becton Dickinson) running CellQuest software and analyzed with FACSDiva software or FlowJo (Tree Star, Ashland, OR).

### Intracellular cytokine staining and *in vivo* neutralization of cytokines

For intracellular cytokine staining single cell suspensions were prepared and cells were incubated with the Cell Stimulation Cocktail (plus protein transport inhibitors, eBioscience) diluted 1:500 in cell culture medium for 4 hours at 37°C. After stimulation cell surface markers were stained as described before. For fixation and permeabilization the Fix&Perm Cell Permeabilization Kit (An Der Grub Bio Research GmbH) was used according to the manufacturer´s specifications. Intracellular cytokines were stained with anti-IFNγ-APC (clone XMG1.2) or anti-IL-17-APC (clone eBio17B7) (eBioscience) diluted 1:100 in reagent B of the kit.

For the *in vivo* neutralization of cytokines, γδ T cell transfers were conducted as described above. In addition neutralizing antibodies against IFNγ (clone XMG1.2), IL-17 (clone 17F3) or their isotype controls (rat IgG1 and mouse IgG1 respectively) were administered. All *in vivo* antibodies were purchased from BioXCell. Mice were injected i.p. with 500 µg of antibody in 200 µl PBS every third day during the course of the experiment, starting one day before the γδ T cell transfer (day 2 after infection).

### Cell depletion

For in vivo depletion of CD4^+^ T cells, mice were injected ip with 250 µg of the monoclonal antibody YTS 191 [48] on day -1, 3 and 8 after infection; for long term depletion with 300 µg antibody on day -1, 3 7, 17 and 27 after infection. For depletion of CD3^+^ T cells 300 µg of YTS 191 were injected on day -4 and 250 µg of the monoclonal antibody 145–2C11 were injected on day -1 and 2 after infection (BioXcell;).

For analysis of whole blood lymphocytes 2 drops of blood were taken from the tail vein in heparinized tubes (Greiner bio-one). Equal volumes of whole blood and buffer (PBS, 2% FCS) containing 2,500 Truecount counting beads (BD Biosciences) and staining antibodies were mixed. BD FACS Lysing solution (BD Biosciences) was added. The mixture was analyzed by flow cytometry.

### Chromium release assay

Purified TCRαβ^+^ or TCRγδ^+^ effector cells were cultured overnight in RPMI-1640 medium with glutamine, penicillin 100 U/ml, streptomycin 0.1 mg/ml, 5 µM β-ME, 10 mM HEPES, 7,5% FCS and 20U/ml interleukin-2. Cytolytic activity was measured by a standard 4-h ^51^Cr release assay with graded numbers of effector cells and with 1,000 target cells per 0.2-ml microwell. Target cells were ^51^Cr-labeled MEF infected with centrifugal enhancement with 0.2 pfu of MCMV per cell in the presence or absence of phosphonoacetic acid for 22 h. CD3ε producing B cell hybridoma cells (145–2C11) were used as targets to measure the total cytolytic potential of an effector cell population by antigen-independent polyclonal signaling via the TCR-CD3 complex [19].

### Cell sorting and adoptive transfer of lymphocytes

Single-cell suspensions of spleens and lymph nodes from at least 6 weeks infected CD8^-/-^JHT mice were stained with antibodies against CD3, CD4, TCRαβ and TCRγδ (see above). CD3^+^, CD4^−^, TCRαβ^+^ or TCRγδ^+^ cells respectively were isolated by fluorescence activated cell sorting using a MoFlo cell sorter (Cytomation) and analyzed for purity. Purity >99% was achieved. Purified cells were either used for chromium release assay or adoptively transferred into the tail or ocular vein of RAG^−/−^ mice 3 days after infection. Absence of contaminating TCRαβ^+^ cells was confirmed by flow cytometry in all animals analyzed for organ titer or survival. Mice with detectable contaminations of TCRαβ^+^ cells were excluded from the analyses.

### BrdU incorporation

Mice were given i.p. injections of 1 mg BrdU. 6 hours after BrdU injections peripheral blood, or cells from spleen, peripheral lymphnodes, liver and lung were harvested and the incorporation of BrdU into the dividing cells’ DNA was determined by the manufacturer’s protocol (FITC BrdU Flow Kit, BD Pharmingen) after surface staining for CD3 and γδTCR.

### High throughput 454 sequencing of VγCγ amplicons from sorted γδ T cells

For high throughput 454 sequencing, γδ T cells from spleen, peripheral (inguinal, brachial, axillary, superficial) lymph nodes, mesenteric lymph nodes, liver and lung of individual mice were sorted by FACS. Two naive mice and two mice four weeks after infection were compared. RNA of γδ T cells from separate organs was isolated with the RNeasy Mini Kit (Qiagen) after homogenizing cells with QIA shredder columns (Qiagen). Before cDNA synthesis with the Transciptor High Fidelity cDNA Synthesis Kit (version 6.0, Roche) volume of the samples was reduced through centrifugation in a SpeedVac centrifuge (Eppendorf). cDNA of individual organs was used for polymerase chain reactions. Primers are listed in S2 Table. For each organ sample five PCRs were carried out and products of every organ were tagged with a specific barcode (multiplex identifier, MID) during PCRs. Reactions contained 4 µl of cDNA template, 3 µl RediLoad (Invitrogen), 1 µl of a 5 µM stock of each primer, 12 µl 5-Prime polymerase mix 2,5x (5 PRIME GmbH) and 9 µl water to obtain a reaction volume of 30 µl.

After heating the reaction mixture for 7 min at 94°C, 38 circles under following conditions were performed: 60 sec at 94°C, 60 sec at 54°C and 30 sec at 72°C. 5 min at 72°C after the last circle allowed final elongation.

10 µl of each reaction were analyzed on an agarose gel and 20 µl were purified with QIAquick columns (PCR purification kit, Qiagen) after pooling the five reactions of one organ. Pools of different organs were adjusted to the same DNA concentration and combined. To reduce the volume, DNA was precipitated with ethanol. Next Generation Sequencing was performed on the Roche 454 platform by MWG eurofins.

After sorting for MID tags for the different samples, individual FASTA files for each individual tissue sample containing 1.000–6.000 high quality reads were analyzed on the IMGT/HighV-QUEST platform [49]. Output files were imported in Microsoft Excel and the Vγ gene usage, CDR3 length and amino acid composition was analyzed for the different samples filtered for sequence reads that contain functional recombinations using the PivotTable function of Excel.

### Statistical analysis

Statistical analysis was performed with Prism 6 (Graph-Pad Software, Inc.). The Mann-Whitney test for the comparison of two groups was used. For the analyses of survival data the Mantel-Cox logrank test was used.

### Ethics statement

The study was performed in strict accordance with German law (Tierschutzgesetz). The protocol was approved by the Committee on Ethics of Animal Experiments at the Bavarian Government (Az. 54–2532.1–57/12 and Az. 54–2532.2–3/08). All efforts were made to minimize animal suffering.

## Supporting Information

S1 Fig CD8^-/-^JHT mice are devoid of CD8^+^ and CD19^+^ cells. Blood of C57BL/6 (left) and CD8^-/-^JHT mice (right) was stained with antibodies against CD8 and CD19 and analyzed by flow cytometry. Cells within the lymphocyte gate are shown.
(TIF)

S2 Fig Administration of antibodies in CD8^-/-^JHT mice and control by flow cytometry. (A) Experimental schedule. Upper row: 250 µg of anti-CD4 antibody YTS 191 were administered at days -1, 3 and 8 p.i. (blue); Lower row: for depletion of CD3 cells 250 µg anti-CD4 antibody was given day -4 p.i. and 250 µg anti-CD3 antibody 145–2C11 was given day -1 and 2 p.i. (green). Days of imaging are marked with an open circle and times of flow cytometric analysis with a red arrow (first experiment: upper row; second experiment: lower row). (B) Representative data at day 13 p.i. obtained by blood cell staining with antibodies against CD4, TCRαβ and TCRγδ followed by flow cytometry (gated on lymphocytes): after anti-CD4 treatment with mab YTS 191 no binding of anti-CD4 antibody GK1.5 to lymphocytes was detected in blood. In blood of CD3-depleted animals no TCRαβ^+^ or TCRγδ^+^ lymphocytes were detected. (C) Detection of γδ T cells in blood of adoptively transferred animals day 12 p.i.
(TIF)

S3 Fig Protective capacity of γδ T cells from naïve and infected wildtype C57Bl/6 mice. Groups of RAG^-/-^ mice were infected with 10^5^ pfu of MCMV157luc and on day 3 of infection 400,000 sorted γδ T cells from the spleen of C57Bl/6 mice were adoptively transferred. Organs were collected on day 18 after infection and viral load per 30 µg organ was determined. The data summarize two independent experiments and are presented as the percentage of virus load compared to a group of RAG^-/-^ mice that received PBS instead of γδ T cells. Box plots represent the median, 25th to 75th percentiles and minimum and maximum values.
(TIF)

S4 Fig Efficient control of MCMV infection in TCRα^-/-^ mice. Groups of RAG^-/-^ and TCRα^-/-^ mice were infected i.v. with 10^6^ pfu of MCMV157luc in which the MCK-2 mutation was repaired. *In vivo* imaging was performed on the days indicated. Images were obtained from a 120sec acquisition. On day 12 after infection Rag-1^-/-^ mice had to be euthanized because of severe sickness.
(TIF)

S5 Fig Efficient control of a secondary MCMV infection in TCRα^-/-^ mice. *In vivo* bioluminescence imaging during a primary (left) and secondary (right) infection. Secondary infection was given 21 days after the primary infection. Mice were infected i.v. with 10^6^ pfu of MCMV157luc in which the MCK-2 mutation was repaired. *In vivo* imaging was performed on the days indicated. Images were obtained from a 120 sec acquisition.
(TIF)

S6 Fig Expression pattern of NKG2D and CD27 on γδ T cells in uninfected mice and 14 days after MCMV infection. CD3^+^ TCRγδ^+^ cells from peripheral blood are gated and analyzed for the surface expression of NKG2D and CD27 by flow cytometry.
(TIF)

S7 Fig Analysis of the Vγ4 and Vγ6 repertoire by high throughput 454 sequencing. Analysis of expanded Vγ4 (left) and Vγ6 (right) clonotypes in infected (d28 post infection, solid bars) and uninfected (open bars) CD8^-/-^JHT mice. The frequency of the most abundant clonotype as defined by identical CDR3 regions is depicted for lung, liver, spleen, peripheral lymph nodes (XLN) and mesenteric lymph nodes (MLN) in 2 individual mice for each group. The CDR3 sequences are presented in the header. A minimum of 125 sequence reads was obtained for all organs and Vγ amplicons.
(TIF)

S1 Table Characteristics of γδ T cells of wild type and CD8^-/-^JHT mice under steady state conditions. (DOCX)

S2 Table Nucleotide sequences of PCR primers used for Vγ-Cγ amplicon generation and 454 high throughput sequencing. (XLSX)

## References

[1] Mach M, Wiegers A-K, Spindler N, Winkler T (2013) Protective Humoral Immunity. In: Reddehase MJ, editor. Cytomegaloviruses: From Molecular Pathogenesis to Intervention. 1 edition ed: Caister Academic Press.

[2] Reddehase MJ (2002) Antigens and immunoevasins: opponents in cytomegalovirus immune surveillance. Nat Rev Immunol 2: 831–844. 10.1038/nri932 12415307

[3] Polić B, Hengel H, Krmpotić A, Trgovcich J, Pavić I, et al (1998) Hierarchical and redundant lymphocyte subset control precludes cytomegalovirus replication during latent infection. J Exp Med 188: 1047–1054. 10.1084/jem.188.6.1047 9743523 PMC2212537

[4] Boeckh M, Geballe AP (2011) Cytomegalovirus: pathogen, paradigm, and puzzle. J Clin Invest 121: 1673–1680. 10.1172/JCI45449 21659716 PMC3083799

[5] Boeckh M, Ljungman P (2009) How we treat cytomegalovirus in hematopoietic cell transplant recipients. Blood 113: 5711–5719. 10.1182/blood-2008-10-143560 19299333 PMC2700312

[6] Kurz S, Steffens HP, Mayer A, Harris JR, Reddehase MJ (1997) Latency versus persistence or intermittent recurrences: evidence for a latent state of murine cytomegalovirus in the lungs. J Virol 71: 2980–2987. 9060657 10.1128/jvi.71.4.2980-2987.1997 PMC191426

[7] Reddehase MJ, Mutter W, Münch K, Bühring HJ, Koszinowski UH (1987) CD8-positive T lymphocytes specific for murine cytomegalovirus immediate-early antigens mediate protective immunity. J Virol 61: 3102–3108. 3041033 10.1128/jvi.61.10.3102-3108.1987 PMC255886

[8] Reddehase MJ, Jonjic S, Weiland F, Mutter W, Koszinowski UH (1988) Adoptive immunotherapy of murine cytomegalovirus adrenalitis in the immunocompromised host: CD4-helper-independent antiviral function of CD8-positive memory T lymphocytes derived from latently infected donors. J Virol 62: 1061–1065. 2828654 10.1128/jvi.62.3.1061-1065.1988 PMC253668

[9] Feuchtinger T, Opherk K, Bethge WA, Topp MS, Schuster FR, et al (2010) Adoptive transfer of pp65-specific T cells for the treatment of chemorefractory cytomegalovirus disease or reactivation after haploidentical and matched unrelated stem cell transplantation. Blood 116: 4360–4367. 10.1182/blood-2010-01-262089 20625005

[10] Jonjic S, Mutter W, Weiland F, Reddehase MJ, Koszinowski UH (1989) Site-restricted persistent cytomegalovirus infection after selective long-term depletion of CD4+ T lymphocytes. J Exp Med 169: 1199–1212. 10.1084/jem.169.4.1199 2564415 PMC2189231

[11] Walton SM, Mandaric S, Torti N, Zimmermann A, Hengel H, et al (2011) Absence of cross-presenting cells in the salivary gland and viral immune evasion confine cytomegalovirus immune control to effector CD4 T cells. PLoS Pathog 7: e1002214 10.1371/journal.ppat.1002214 21901102 PMC3161985

[12] Hammoud B, Schmueck M, Fischer AM, Fuehrer H, Park S-J, et al (2013) HCMV-specific T-cell therapy: do not forget supply of help. J Immunother 36: 93–101. 10.1097/CJI.0b013e31827b87cc 23377662

[13] Wirtz N, Schader SI, Holtappels R, Simon CO, Lemmermann NAW, et al (2008) Polyclonal cytomegalovirus-specific antibodies not only prevent virus dissemination from the portal of entry but also inhibit focal virus spread within target tissues. Med Microbiol Immunol 197: 151–158. 10.1007/s00430-008-0095-0 18365251

[14] Klenovsek K, Weisel F, Schneider A, Appelt U, Jonjic S, et al (2007) Protection from CMV infection in immunodeficient hosts by adoptive transfer of memory B cells. Blood 110: 3472–3479. 10.1182/blood-2007-06-095414 17656648

[15] Jonjic S, Pavić I, Polić B, Crnković I, Lucin P, et al (1994) Antibodies are not essential for the resolution of primary cytomegalovirus infection but limit dissemination of recurrent virus. J Exp Med 179: 1713–1717. 10.1084/jem.179.5.1713 8163949 PMC2191473

[16] Farrell HE, Shellam GR (1991) Protection against murine cytomegalovirus infection by passive transfer of neutralizing and non-neutralizing monoclonal antibodies. J Gen Virol 72 (Pt 1): 149–156. 10.1099/0022-1317-72-1-149 1846643

[17] Jonjic S, Pavić I, Lucin P, Rukavina D, Koszinowski UH (1990) Efficacious control of cytomegalovirus infection after long-term depletion of CD8+ T lymphocytes. J Virol 64: 5457–5464. 1976821 10.1128/jvi.64.11.5457-5464.1990 PMC248597

[18] French AR, Pingel JT, Wagner M, Bubic I, Yang L, et al (2004) Escape of mutant double-stranded DNA virus from innate immune control. Immunity 20: 747–756. 10.1016/j.immuni.2004.05.006 15189739

[19] Holtappels R, Podlech J, Grzimek NK, Thomas D, Pahl-Seibert MF, et al (2001) Experimental preemptive immunotherapy of murine cytomegalovirus disease with CD8 T-cell lines specific for ppM83 and pM84, the two homologs of human cytomegalovirus tegument protein ppUL83 (pp65). J Virol 75: 6584–6600. 10.1128/JVI.75.14.6584-6600.2001 11413326 PMC114382

[20] Jordan S, Krause J, Prager A, Mitrovic M, Jonjic S, et al (2011) Virus Progeny of Murine Cytomegalovirus Bacterial Artificial Chromosome pSM3fr Show Reduced Growth in Salivary Glands due to a Fixed Mutation of MCK-2. J Virol 85: 10346–10353. 10.1128/JVI.00545-11 21813614 PMC3196435

[21] Vantourout P, Hayday A (2013) Six-of-the-best: unique contributions of γδ T cells to immunology. Nat Rev Immunol 13: 88–100. 10.1038/nri3384 23348415 PMC3951794

[22] Ribot JC, Debarros A, Pang DJ, Neves JF, Peperzak V, et al (2009) CD27 is a thymic determinant of the balance between interferon-gamma- and interleukin 17-producing gammadelta T cell subsets. Nat Immunol 10: 427–436. 10.1038/ni.1717 19270712 PMC4167721

[23] Carding SR, Egan PJ (2002) Gammadelta T cells: functional plasticity and heterogeneity. Nat Rev Immunol 2: 336–345. 10.1038/nri797 12033739

[24] Kamphuis E, Junt T, Waibler Z, Förster R, Kalinke U (2006) Type I interferons directly regulate lymphocyte recirculation and cause transient blood lymphopenia. Blood 108: 3253–3261. 10.1182/blood-2006-06-027599 16868248

[25] Jamieson AM, Diefenbach A, McMahon CW, Xiong N, Carlyle JR, et al (2002) The role of the NKG2D immunoreceptor in immune cell activation and natural killing. Immunity 17: 19–29. 10.1016/S1074-7613(02)00333-3 12150888

[26] Gravestein LA, Nieland JD, Kruisbeek AM, Borst J (1995) Novel mAbs reveal potent co-stimulatory activity of murine CD27. Int Immunol 7: 551–557. 10.1093/intimm/7.4.551 7547681

[27] Déchanet J, Merville P, Lim A, Retière C, Pitard V, et al (1999) Implication of gammadelta T cells in the human immune response to cytomegalovirus. J Clin Invest 103: 1437–1449. 10.1172/JCI5409 10330426 PMC408467

[28] Heilig JS, Tonegawa S (1986) Diversity of murine gamma genes and expression in fetal and adult T lymphocytes. Nature 322: 836–840. 10.1038/322836a0 2943999

[29] Balthesen M, Messerle M, Reddehase MJ (1993) Lungs are a major organ site of cytomegalovirus latency and recurrence. J Virol 67: 5360–5366. 8394453 10.1128/jvi.67.9.5360-5366.1993 PMC237936

[30] Turchinovich G, Pennington DJ (2011) T cell receptor signalling in γδ cell development: strength isn&apos;t everything. Trends Immunol 32: 567–573. 10.1016/j.it.2011.09.005 22056207

[31] Sun JC, Beilke JN, Lanier LL (2009) Adaptive immune features of natural killer cells. Nature 457: 557–561. 10.1038/nature07665 19136945 PMC2674434

[32] Scheper W, van Dorp S, Kersting S, Pietersma F, Lindemans C, et al (2013) γδT cells elicited by CMV reactivation after allo-SCT cross-recognize CMV and leukemia. Leukemia 27: 1328–1338. 10.1038/leu.2012.374 23277330

[33] Dieli F, Poccia F, Lipp M, Sireci G, Caccamo N, et al (2003) Differentiation of effector/memory Vdelta2 T cells and migratory routes in lymph nodes or inflammatory sites. J Exp Med 198: 391–397. 10.1084/jem.20030235 12900516 PMC2194087

[34] Carding SR, Allan W, Kyes S, Hayday A, Bottomly K, et al (1990) Late dominance of the inflammatory process in murine influenza by gamma/delta + T cells. J Exp Med 172: 1225–1231. 10.1084/jem.172.4.1225 2145388 PMC2188600

[35] Alejenef A, Pachnio A, Halawi M, Christmas SE, Moss PA, et al (2014) Cytomegalovirus drives Vdelta2neg gammadelta T cell inflation in many healthy virus carriers with increasing age. Clin Exp Immunol 176: 418–428. 10.1111/cei.12297 24547915 PMC4008987

[36] Willcox CR, Pitard V, Netzer S, Couzi L, Salim M, et al (2012) Cytomegalovirus and tumor stress surveillance by binding of a human γδ T cell antigen receptor to endothelial protein C receptor. Nat Immunol 13: 872–879. 10.1038/ni.2394 22885985

[37] Dyugovskaya L, Hirsh M, Ginsburg H (2003) Phenotypic profile and functional characterization of rat lymph node-derived gammadelta T cells: implication in the immune response to cytomegalovirus. Immunology 108: 129–136. 10.1046/j.1365-2567.2003.01568.x 12562320 PMC1782877

[38] Ninomiya T, Takimoto H, Matsuzaki G, Hamano S, Yoshida H, et al (2000) Vgamma1+ gammadelta T cells play protective roles at an early phase of murine cytomegalovirus infection through production of interferon-gamma. Immunology 99: 187–194. 10.1046/j.1365-2567.2000.00938.x 10692035 PMC2327158

[39] Halary F, Pitard V, Dlubek D, Krzysiek R, de la Salle H, et al (2005) Shared reactivity of Vdelta2neg gamma delta T cells against cytomegalovirus-infected cells and tumor intestinal epithelial cells. J Exp Med 201: 1567–1578. 10.1084/jem.20041851 15897274 PMC2212929

[40] Lafarge X, Merville P, Cazin MC, Bergé F, Potaux L, et al (2001) Cytomegalovirus infection in transplant recipients resolves when circulating gammadelta T lymphocytes expand, suggesting a protective antiviral role. J Infect Dis 184: 533–541. 10.1086/322843 11494158

[41] Knight A, Madrigal AJ, Grace S, Sivakumaran J, Kottaridis P, et al (2010) The role of Vδ2-negative γδ T cells during cytomegalovirus reactivation in recipients of allogeneic stem cell transplantation. Blood 116: 2164–2172. 10.1182/blood-2010-01-255166 20576814

[42] Vermijlen D, Brouwer M, Donner C, Liesnard C, Tackoen M, et al (2010) Human cytomegalovirus elicits fetal gammadelta T cell responses in utero. J Exp Med 207: 807–821. 10.1084/jem.20090348 20368575 PMC2856038

[43] Handgretinger R (2012) Negative depletion of CD3(+) and TcRαβ(+) T cells. Curr Opin Hematol 19: 434–439. 10.1097/MOH.0b013e3283582340 22914586

[44] Godder KT, Henslee-Downey PJ, Mehta J, Park BS, Chiang K-Y, et al (2007) Long term disease-free survival in acute leukemia patients recovering with increased gammadelta T cells after partially mismatched related donor bone marrow transplantation. Bone Marrow Transplant 39: 751–757. 10.1038/sj.bmt.1705650 17450185

[45] Chen J, Trounstine M, Alt FW, Young F, Kurahara C, et al (1993) Immunoglobulin gene rearrangement in B cell deficient mice generated by targeted deletion of the JH locus. Int Immunol 5: 647–656. 10.1093/intimm/5.6.647 8347558

[46] Fung-Leung WP, Schilham MW, Rahemtulla A, Kündig TM, Vollenweider M, et al (1991) CD8 is needed for development of cytotoxic T cells but not helper T cells. Cell 65: 443–449. 10.1016/0092-8674(91)90462-8 1673361

[47] Podlech J, Holtappels R, Grzimek NKA, Reddehase MJ (2002) Animal models: Murine cytomegalovirus. In: Stefan H. E KDK, editor. Methods in Microbiology: Academic Press pp. 493–525.

[48] Cobbold SP, Jayasuriya A, Nash A, Prospero TD, Waldmann H (1984) Therapy with monoclonal antibodies by elimination of T-cell subsets in vivo. Nature 312: 548–551. 10.1038/312548a0 6150440

[49] Alamyar E, Duroux P, Lefranc MP, Giudicelli V (2012) IMGT((R)) tools for the nucleotide analysis of immunoglobulin (IG) and T cell receptor (TR) V-(D)-J repertoires, polymorphisms, and IG mutations: IMGT/V-QUEST and IMGT/HighV-QUEST for NGS. Methods Mol Biol 882: 569–604. 10.1007/978-1-61779-842-9_32 22665256

