# Supplementary figures and images for: Control of Murine Cytomegalovirus Infection by γδ T Cells
Source: PLoS Pathog. 2015 Feb 6;11(2):e1004481. doi: 10.1371/journal.ppat.1004481 (PMC4450058; doi:10.1371/journal.ppat.1004481)

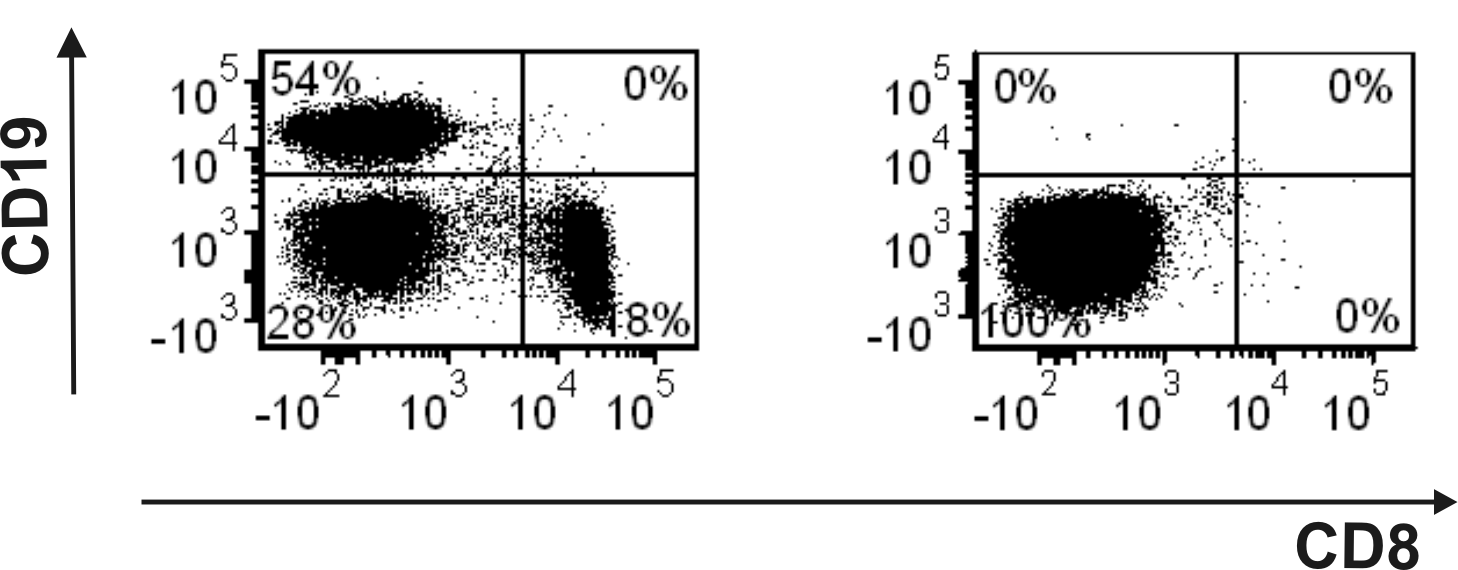

Supplement: S1 Fig — Blood of C57BL/6 (left) and CD8-/-JHT mice (right) was stained with antibodies against CD8 and CD19 and analyzed by flow cytometry. Cells within the lymphocyte gate are shown. (TIF) [file ppat.1004481.s001.tif]

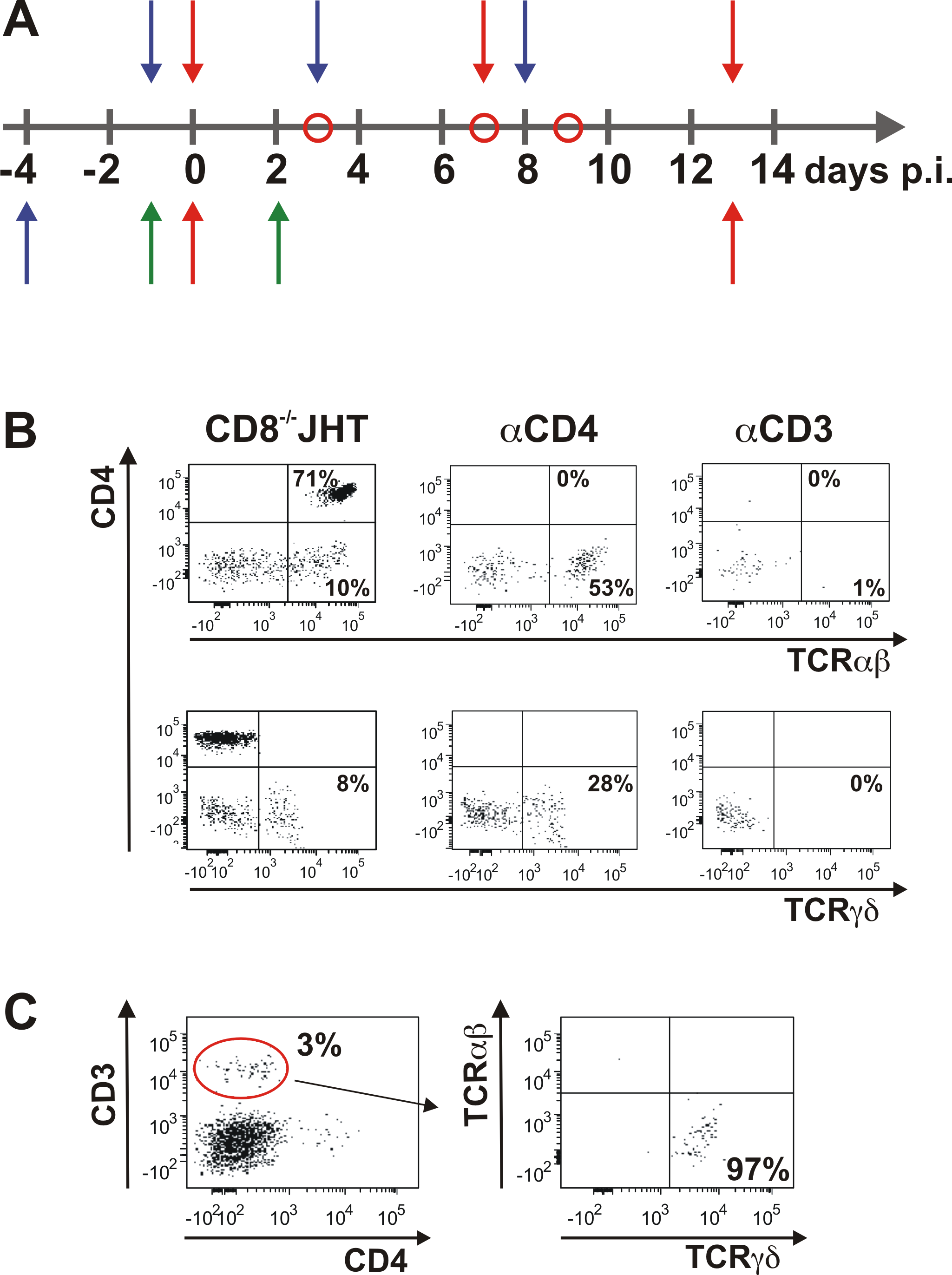

Supplement: S2 Fig — (A) Experimental schedule. Upper row: 250 µg of anti-CD4 antibody YTS 191 were administered at days -1, 3 and 8 p.i. (blue); Lower row: for depletion of CD3 cells 250 µg anti-CD4 antibody was given day -4 p.i. and 250 µg anti-CD3 antibody 145–2C11 was given day -1 and 2 p.i. (green). Days of imaging are marked with an open circle and times of flow cytometric analysis with a red arrow (first experiment: upper row; second experiment: lower row). (B) Representative data at day 13 p.i. obtained by blood cell staining with antibodies against CD4, TCRαβ and TCRγδ followed by flow cytometry (gated on lymphocytes): after anti-CD4 treatment with mab YTS 191 no binding of anti-CD4 antibody GK1.5 to lymphocytes was detected in blood. In blood of CD3-depleted animals no TCRαβ+ or TCRγδ+ lymphocytes were detected. (C) Detection of γδ T cells in blood of adoptively transferred animals day 12 p.i. (TIF) [file ppat.1004481.s002.tif]

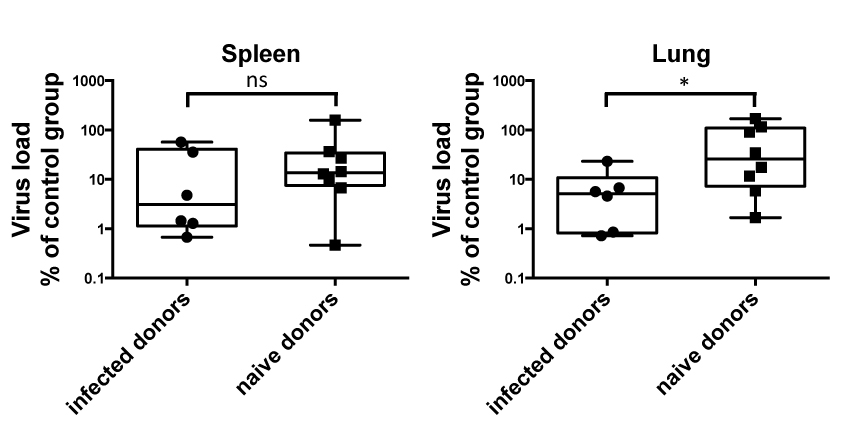

Supplement: S3 Fig — Groups of RAG-/- mice were infected with 105 pfu of MCMV157luc and on day 3 of infection 400,000 sorted γδ T cells from the spleen of C57Bl/6 mice were adoptively transferred. Organs were collected on day 18 after infection and viral load per 30 µg organ was determined. The data summarize two independent experiments and are presented as the percentage of virus load compared to a group of RAG-/- mice that received PBS instead of γδ T cells. Box plots represent the median, 25th to 75th percentiles and minimum and maximum values. (TIF) [file ppat.1004481.s003.tif]

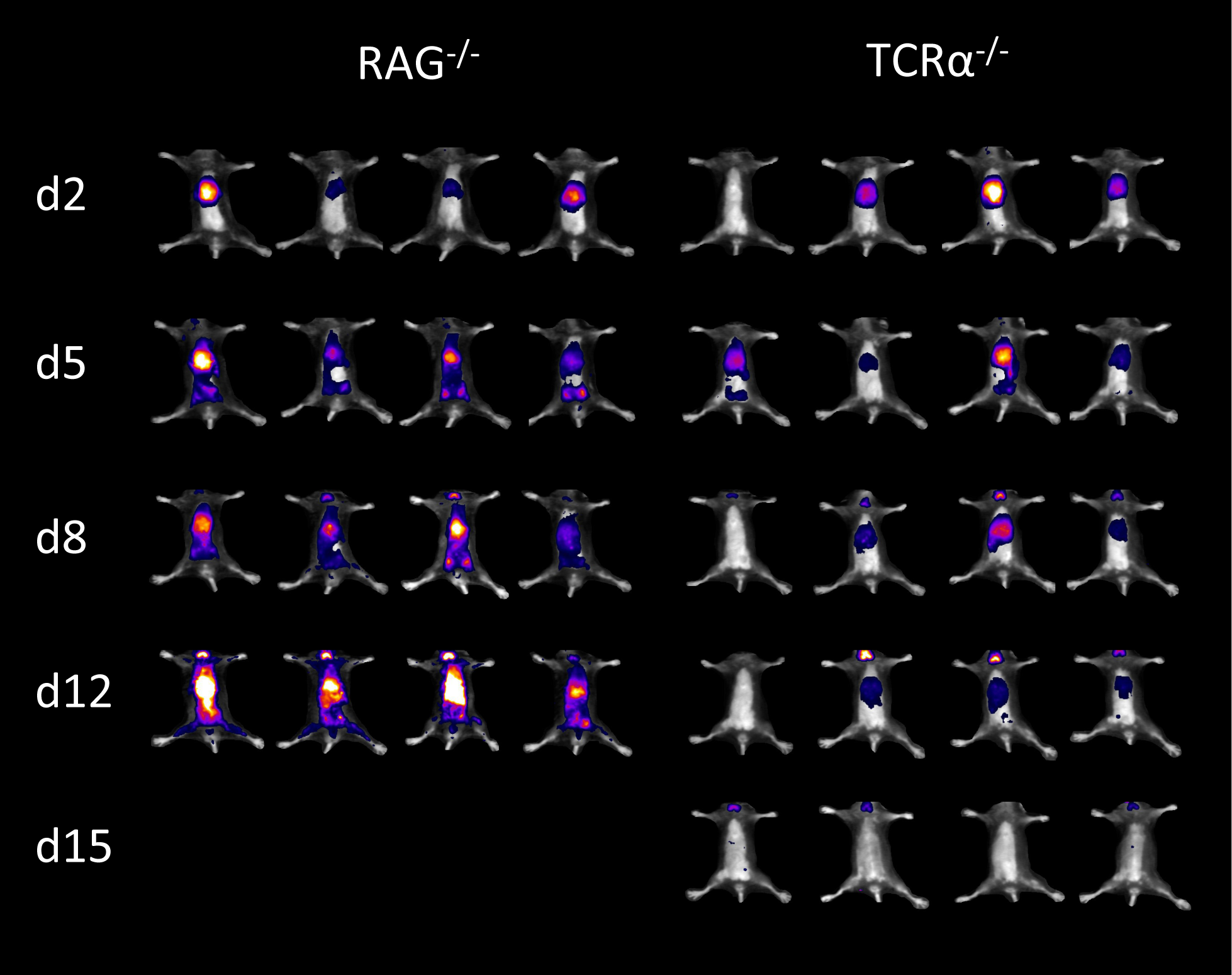

Supplement: S4 Fig — Groups of RAG-/- and TCRα-/- mice were infected i.v. with 106 pfu of MCMV157luc in which the MCK-2 mutation was repaired. In vivo imaging was performed on the days indicated. Images were obtained from a 120sec acquisition. On day 12 after infection Rag-1-/- mice had to be euthanized because of severe sickness. (TIF) [file ppat.1004481.s004.tif]

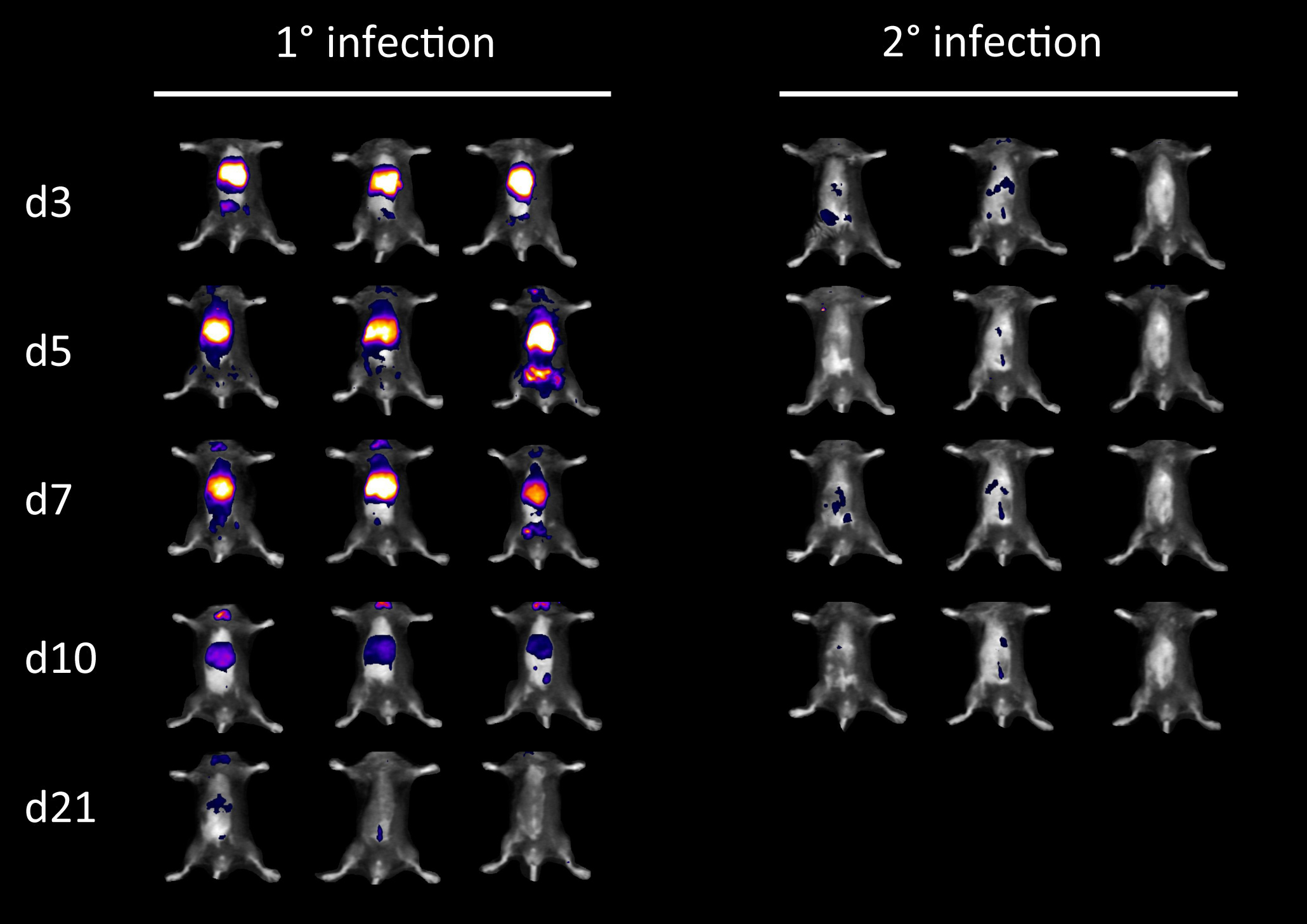

Supplement: S5 Fig — In vivo bioluminescence imaging during a primary (left) and secondary (right) infection. Secondary infection was given 21 days after the primary infection. Mice were infected i.v. with 106 pfu of MCMV157luc in which the MCK-2 mutation was repaired. In vivo imaging was performed on the days indicated. Images were obtained from a 120 sec acquisition. (TIF) [file ppat.1004481.s005.tif]

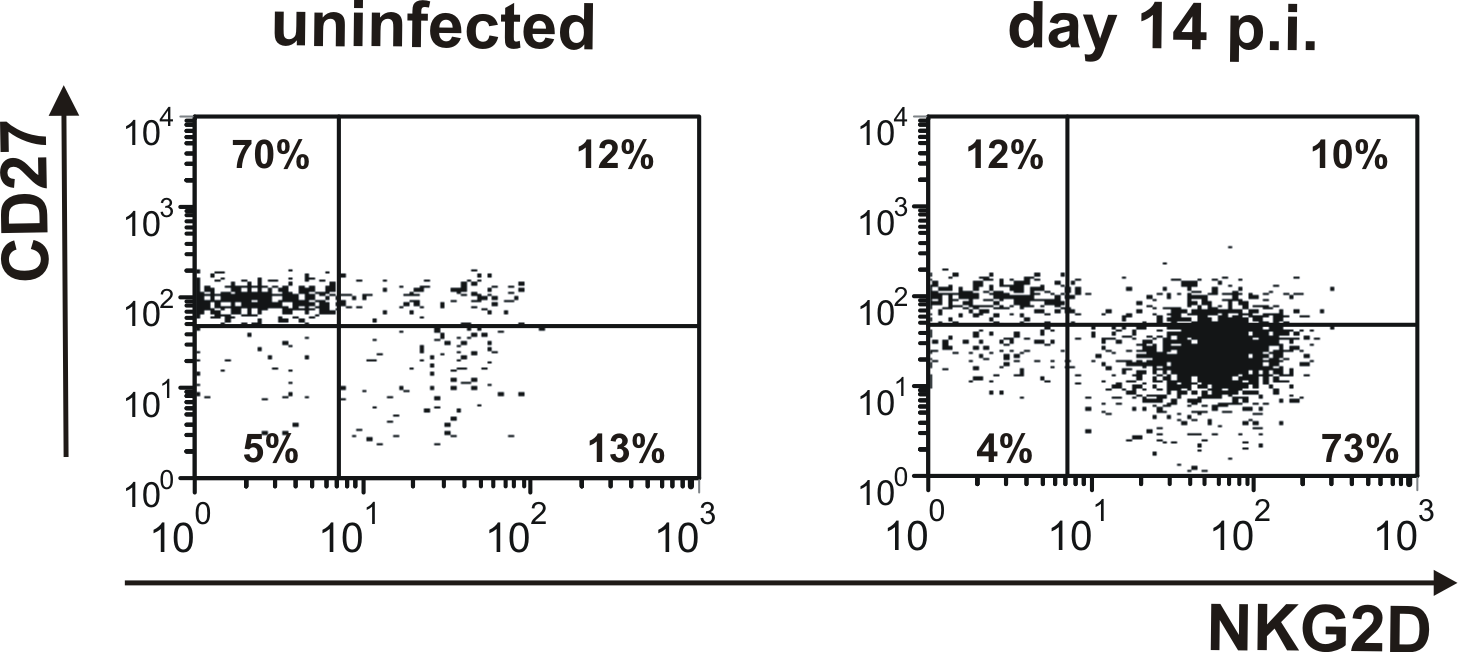

Supplement: S6 Fig — CD3+ TCRγδ+ cells from peripheral blood are gated and analyzed for the surface expression of NKG2D and CD27 by flow cytometry. (TIF) [file ppat.1004481.s006.tif]
