# Supplementary material for: Control of Murine Cytomegalovirus Infection by γδ T Cells
Source: PLoS Pathog. 2015 Feb 6;11(2):e1004481. doi: 10.1371/journal.ppat.1004481 (PMC4450058; doi:10.1371/journal.ppat.1004481)
Supplement: S1 Table — (DOCX) [file ppat.1004481.s008.docx]

**Table S1: Characteristics of γδT cells of wild type and CD8^-/-^JHT mice under steady state conditions**

|  | **C57BL/6** | **CD8^-/-^JHT** |
| --- | --- | --- |
| **Absolute cell numbers** |  |  |
| Blood | 25±10,03 | 52,75±20,45 |
| Spleen | 4,33±0,82 | 1,61±0,53 |
| Liver | 0,36±0,12 | 1,97±2,48 |
| Lung | 0,36±0,14 | 0,4±0,15 |
|  |  |  |
| **Subsets (%)** |  |  |
| ***NKG2D*** |  |  |
| Blood | 23,75±7,04 | 32,9±8,48 |
| Spleen | 30,75±6,2 | 39,43±3,86 |
| Liver | 57,4±3,6 | 52,55±3,76 |
| Lung | 43,53±7,97 | 44,23±8,94 |
|  |  |  |
| ***CD44*** |  |  |
| Blood | 52,65±7,31 | 67,3±11,82 |
| Spleen | 65,85±1,65 | 78,78±1,33 |
| Liver | 82,8±2 | 91,33±7,05 |
| Lung | 73,28±5,21 | 77±4,27 |
|  |  |  |
| ***CD27high*** |  |  |
| Blood | 78,28±13,09 | 76,05±13,28 |
| Spleen | 75,83±7,56 | 63,13±3,64 |
| Liver | 28,2±2,33 | 18,23±4,21 |
| Lung | 45,33±8,8 | 51,5±5,9 |

Absolute cell numbers and percentages of indicated subsets for C57BL/6 (wild type) and CD8^-/-^JHT mice; values are per μl blood and x10^5^ for spleen, liver and lung; mean and standard deviation of four mice are presented
